# Supplementary material for: Modeling metastatic progression from cross-sectional cancer genomics data
Source: Bioinformatics. 2024 Jun 28;40(Suppl 1):i140–50. doi: 10.1093/bioinformatics/btae250 (PMC11245855; doi:10.1093/bioinformatics/btae250)
Supplement: btae250_Supplementary_Data [file btae250_supplementary_data.pdf]

Supplement to: Modeling metastatic progression from cross-sectional cancer genomics data

Kevin Rupp Andreas Lösch Y. Linda Hu Chenxi Nie Rudolf Schill
Maren Klever Simon Pfahler Lars Grasedyck Tilo Wettig
Niko Beerenwinkel Rainer Spang

1 Sum of tensor product formulations

Here we provide formulas for the transition rate matrices $Q_0, Q_\mathrm{P}, Q_\mathrm{M}, Q_\mathrm{S}$ as sums of tensor products. We use "$\cdot$" to denote 0.

$$Q_0 = \sum_{i=1}^{n} \left(\bigotimes_{j=1}^{i} \begin{pmatrix} 1 & \cdot & \cdot & \cdot \\ \cdot & \cdot & \cdot & \cdot \\ \cdot & \cdot & \cdot & \cdot \\ \cdot & \cdot & \cdot & \Theta_{i,j} \end{pmatrix} \otimes \begin{pmatrix} -\Theta_{i,i} & \cdot & \cdot & \cdot \\ \cdot & \cdot & \cdot & \cdot \\ \cdot & \cdot & \cdot & \cdot \\ \Theta_{i,i} & \cdot & \cdot & \cdot \end{pmatrix} \otimes \bigotimes_{j=i+1}^{n} \begin{pmatrix} 1 & \cdot & \cdot & \cdot \\ \cdot & \cdot & \cdot & \cdot \\ \cdot & \cdot & \cdot & \cdot \\ \cdot & \cdot & \cdot & \Theta_{i,j} \end{pmatrix} \right) \otimes \begin{pmatrix} 1 & \cdot \\ \cdot & \cdot \end{pmatrix} \tag{1}$$

$$Q_\mathrm{S} = \Theta_{n+1,n+1} \bigotimes_{j=1}^{n} \begin{pmatrix} 1 & \cdot & \cdot & \cdot \\ \cdot & \cdot & \cdot & \cdot \\ \cdot & \cdot & \cdot & \cdot \\ \cdot & \cdot & \cdot & \Theta_{n+1,j} \end{pmatrix} \otimes \begin{pmatrix} -1 & \cdot \\ 1 & \cdot \end{pmatrix} \tag{2}$$

$$Q_\mathrm{P} = \sum_{i=1}^{n} \left(\bigotimes_{j=1}^{i} \begin{pmatrix} 1 & \cdot & \cdot & \cdot \\ \cdot & \Theta_{i,j} & \cdot & \cdot \\ \cdot & \cdot & 1 & \cdot \\ \cdot & \cdot & \cdot & \Theta_{i,j} \end{pmatrix} \otimes \begin{pmatrix} -\Theta_{i,i} & \cdot & \cdot & \cdot \\ \Theta_{i,i} & \cdot & \cdot & \cdot \\ \cdot & \cdot & -\Theta_{i,j} & \cdot \\ \cdot & \cdot & \Theta_{i,j} & \cdot \end{pmatrix} \otimes \bigotimes_{j=i+1}^{n} \begin{pmatrix} 1 & \cdot & \cdot & \cdot \\ \cdot & \Theta_{i,j} & \cdot & \cdot \\ \cdot & \cdot & 1 & \cdot \\ \cdot & \cdot & \cdot & \Theta_{i,j} \end{pmatrix} \right) \otimes \begin{pmatrix} \cdot & \cdot \\ \cdot & 1 \end{pmatrix} \tag{3}$$

$$Q_\mathrm{M} = \sum_{i=1}^{n} \left(\bigotimes_{j=1}^{i} \begin{pmatrix} 1 & \cdot & \cdot & \cdot \\ \cdot & 1 & \cdot & \cdot \\ \cdot & \cdot & \Theta_{i,j} & \cdot \\ \cdot & \cdot & \cdot & \Theta_{i,j} \end{pmatrix} \otimes \begin{pmatrix} -\Theta_{i,i} & \cdot & \cdot & \cdot \\ \cdot & -\Theta_{i,j} & \cdot & \cdot \\ \Theta_{i,j} & \cdot & \cdot & \cdot \\ \cdot & \Theta_{i,j} & \cdot & \cdot \end{pmatrix} \otimes \bigotimes_{j=i+1}^{n} \begin{pmatrix} 1 & \cdot & \cdot & \cdot \\ \cdot & 1 & \cdot & \cdot \\ \cdot & \cdot & \Theta_{i,j} & \cdot \\ \cdot & \cdot & \cdot & \Theta_{i,j} \end{pmatrix} \otimes \begin{pmatrix} \cdot & \cdot \\ \cdot & \Theta_{i,n+1} \end{pmatrix} \right) \tag{4}$$

Here we additionally provide formulas for the diagonal matrices U_P and U_M, that hold the observation rates, as sums of tensor products:

$$U_\mathrm{P} := \bigotimes_{i=1}^{n} \begin{pmatrix} 1 & \cdot & \cdot & \cdot \\ \cdot & \Omega_{\mathrm{P}\,i} & \cdot & \cdot \\ \cdot & \cdot & 1 & \cdot \\ \cdot & \cdot & \cdot & \Omega_{\mathrm{P}\,i} \end{pmatrix} \otimes \begin{pmatrix} \Omega_{\mathrm{P}\,n+1} & \cdot \\ \cdot & \Omega_{\mathrm{P}\,n+1} \end{pmatrix} \tag{5}$$

$$U_\mathrm{M} := \bigotimes_{i=1}^{n} \begin{pmatrix} 1 & \cdot & \cdot & \cdot \\ \cdot & 1 & \cdot & \cdot \\ \cdot & \cdot & \Omega_{\mathrm{M}\,i} & \cdot \\ \cdot & \cdot & \cdot & \Omega_{\mathrm{M}\,i} \end{pmatrix} \otimes \begin{pmatrix} \cdot & \cdot \\ \cdot & \Omega_{\mathrm{M}\,n+1} \end{pmatrix} \tag{6}$$

2 Parameter recovery

We conducted a simulation study to investigate how well metMHN can recover model parameters from observational data. We first generated a ground truth model (shown in Figure S1) to use for all subsequent simulations. For this we inferred a metMHN-model on the LUAD-dataset. We did not use the full set of events, instead we restricted ourselves to the set of events containing only the 20 point mutations. We simulated 100 different datasets according to the ground truth model using the stochastic simulation algorithm [4]. Each synthetic dataset contained 5000 data points. As outlined in the main text's data preparation section, only a small fraction of metastasized tumors in the LUAD dataset have known profiles for both primary tumor and metastasis. Therefore, in order to test metMHN's capabilities in a more realistic scenario we discarded either the primary tumor profile or the metastasis profile in 90% of the metastasized samples. Specifically, we discarded the primary tumor profile in 50% of the metastasized samples, and additionally excluded the metastasis profile in 40% of the remaining metastasized samples. Thus we kept both profiles in only 10% of the metastasized cases. Then we fit an metMHN-model on each synthetic dataset. We used the same penalty weight each time, which we determined beforehand once in a 5 fold-cross validation. Figure S2 shows the average of all inferred models and Figure S3 shows the variance over all inferred effects.

A comparison between the ground truth model Figure S1 and the averaged Figure S2 shows that overall metMHN can recover the ground truth parameters quite well. Notably, metMHN model demonstrates high accuracy in recovering the most prominent inter-genomic effects, such as the interactions between EGFR and KRAS or between KEAP1 and STK11. However, the recovery performance declines when it comes to less prominent inter-genomic effects. In these cases, the model tends to underestimate the effect strengths, as evidenced by the effect of STK11 on SMARCA4 and the effect of KEAP1 on the Seeding event. Particularly challenging are weak pairwise effects between mutations occurring at low base rates, as observed in the effects between PTPRT and PTPRD, as well as PTPRT and EPHA3. Curiously the inferred logarithmic base rates appear to be globally shifted by an offset of around $0.4 - 0.5$ as compared to the ground truth models. Additionally the variance of the effects of the inferred models (shown in Figure S3) are small compared to the effect strengths, demonstrating consistent estimators with low variability. Overall we conclude that metMHN can (at least) approximately recover most of the ground-truth effects in this realistic simulation setting.

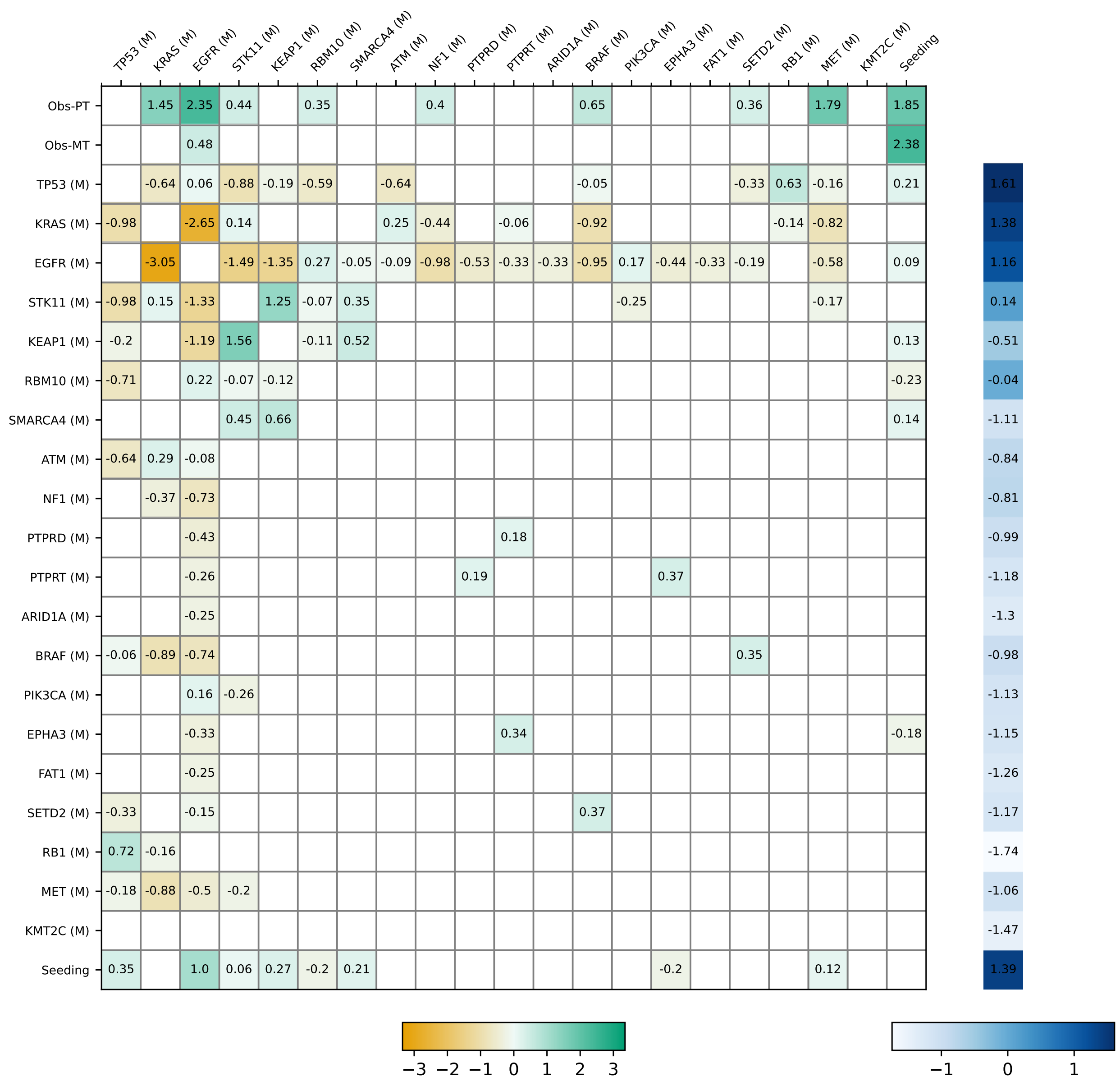

Figure S1: Ground truth model with logarithmic entries used for simulations in the parameter recovery analysis. The effects of mutations on the observation of the primary tumor and the effects of mutations on the observation of the metastasis are plotted in the first two rows, the remaining matrix shows the interaction effects among mutations. The base rates are extracted and plotted separately on the right.

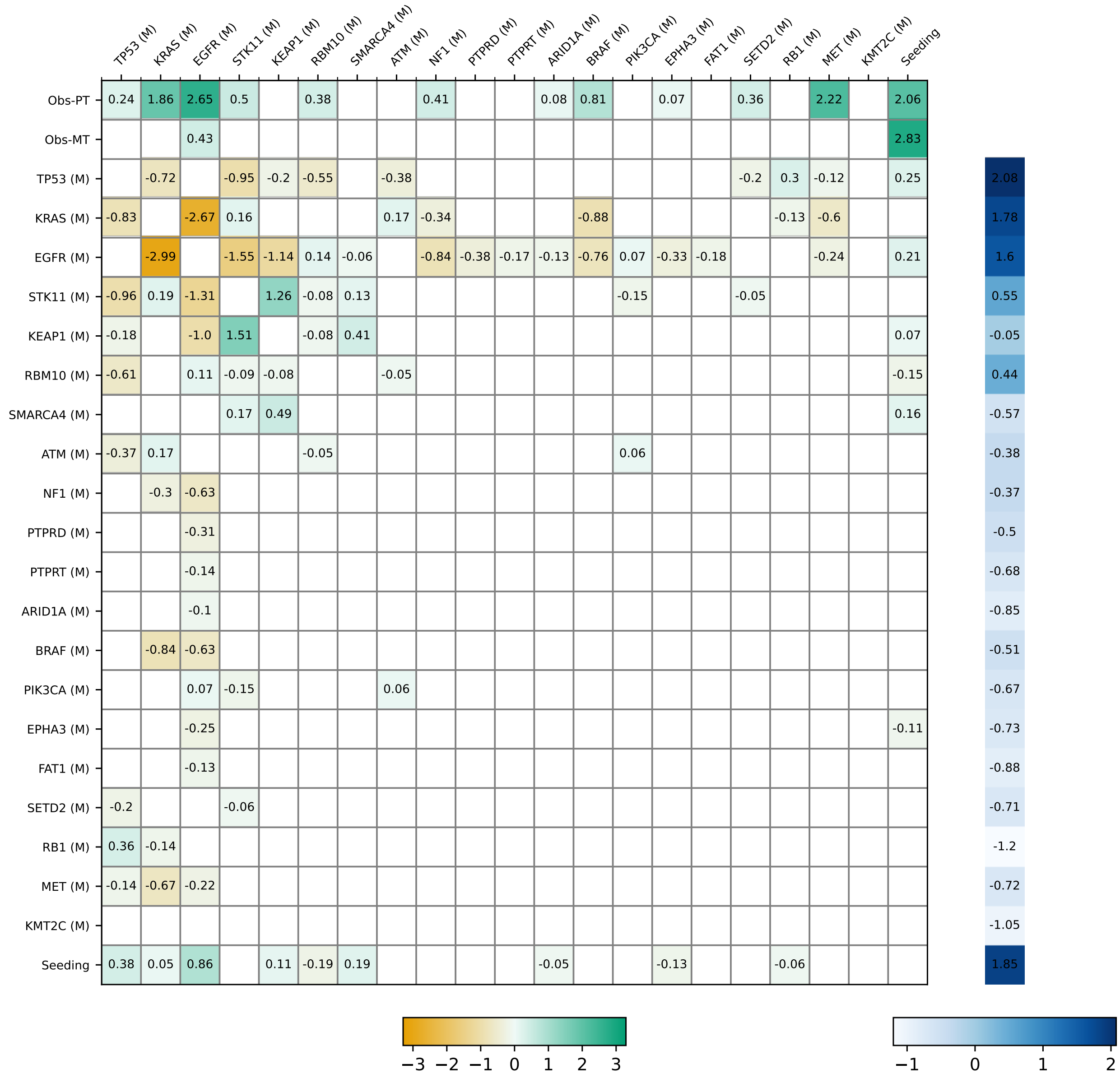

Figure S2: Average of inferred models, estimated from 100 repetitions of randomly generated datasets containing 5000 data points each. All effects are logarithmic. The inferred effects of mutations on the observation of the primary tumor and the effects of mutations on the observation of the metastasis are plotted in the first two rows, the remaining matrix shows the interaction effects among mutations. The base rates are extracted and plotted separately on the right.

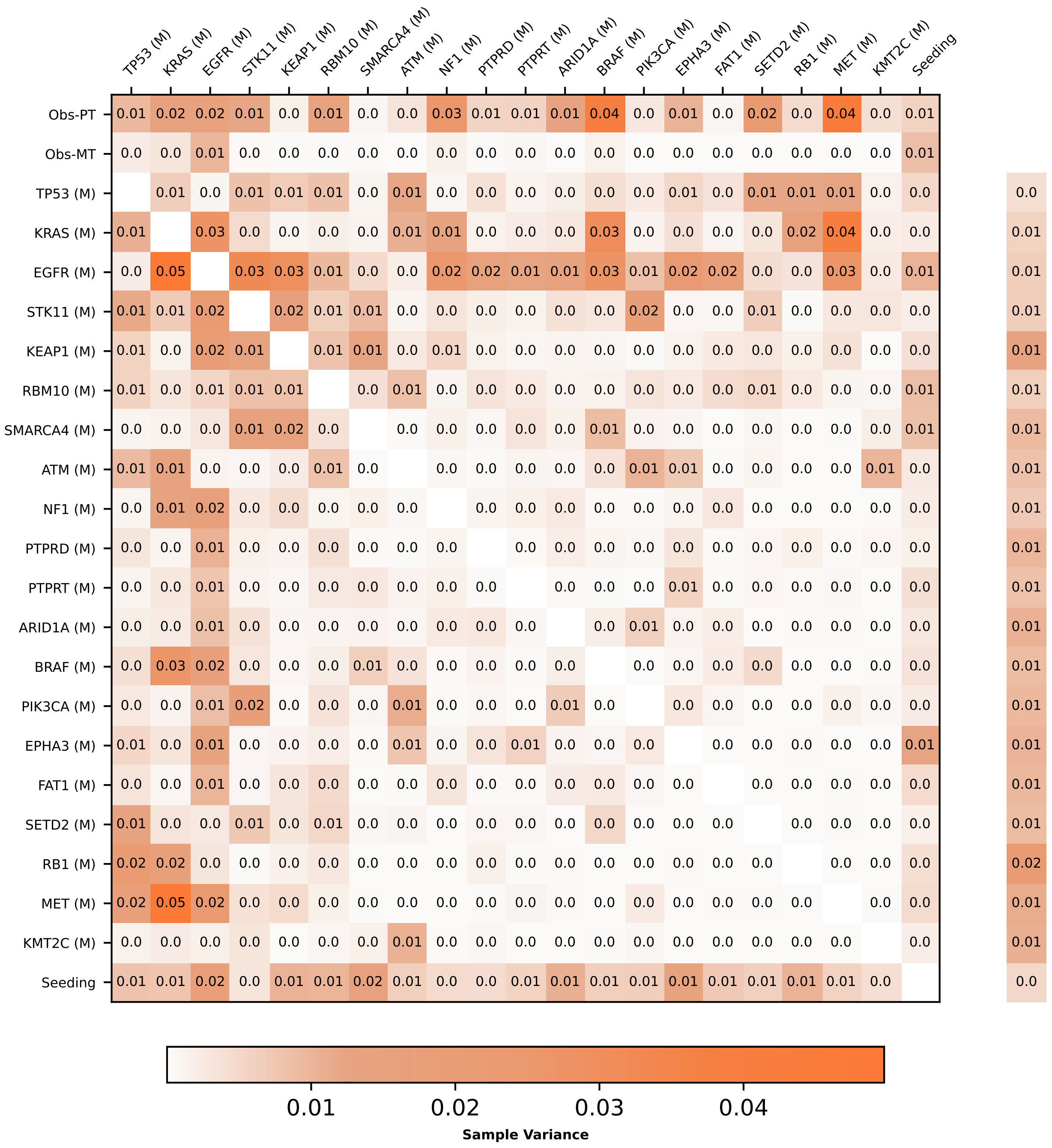

Figure S3: Variance of inferred models, estimated from 100 repetitions of randomly generated datasets containing 5000 data points each. The variances of the inferred effects of mutations on the observation of the primary tumor and on the observation of the metastasis are plotted in the first two rows, the remaining matrix shows the variances of the inferred interaction effects among mutations. The variances of the inferred base rates are extracted and plotted separately on the right.

3 Comparison between metMHN inferences and phylogenetic orderings

3.1 Methods

We aimed to compare trends in driver mutational event orderings derived from metMHN with those obtainable from phylogenetic analyses. For the phylogenetic side of the comparison, we obtained the results on 214 TCGA LUAD primary tumors published in [5]. In this work, PhyloWGS [2] was used to infer clonal phylogenetic trees of single-sample whole-exome sequenced primary tumors. For each of the 214 samples for which phylogenetic inferences were available, we chose the one PhyloWGS run that produced the output with the greatest likelihood. Considering those 214 phylogenetic trees, we next focused on the 190 pairs of mutational (driver) events which we also included in our analysis. For each of these pairs, we analysed each tree whether both events were present, and if so, whether they had been assigned to the same clone or not. If both were present but in different clones, we checked for parent-child relationships while discounting cases where the two clones did not arise sequentially. Thus, for each of the 190 event pairs, we counted the instances in which event A was inferred to be in a clone with a parental relationship to the clone with event B (i.e., A was acquired before B) and vice versa (B was acquired before A). We further counted how often A and B were assigned to the same clone. We then restricted the 190 event pairs to those 14 event pairs in which a parent-child relationship was inferred at least 4 times (in different patients). Of those 14 pairs, we assessed ordering trends by comparing the counts of ‘A before B’ with ‘B before A’ and ‘both in the same clone’.

To assess trends in pairwise event ordering encoded in the metMHN model, we first simulated the progression of 1,000,000 synthetic patients. Of those patients, we only considered the event orderings in the primary tumor part (analogous the PhyloWGS analysis). For each event pair we then assessed the percentage of event orderings in which A was acquired before B and vice versa.

For each of the 14 comparable event pairs, we present here the more frequent ordering as indicated by PhyloWGS, the counts of discordant orderings and same-clone relationships as inferred by PhyloWGS, and compare these to the percentage of metMHN-derived event orderings which are in concordance with the ordering in question (see Figure S4).

3.2 Results and Discussion

The inferences made by PhyloWGS roughly align with metMHN, in particular for multiple event pairs where TP53 mutations are followed by relatively rare secondary driver mutations, such as KMT2C and PTPRD. Interestingly, although TP53 mutation is the most frequent event in the analysis, both methods agree in that they are usually preceded by substantially rarer mutations in KRAS and EGFR. However, there is also disagreement between the orderings inferred by PhyloWGS and metMHN, particularly in cases where PhyloWGS places a relatively rare event such as PIK3CA mutations before TP53 mutations, or in cases where both events are relatively rare (e.g., KEAP1-KMT2C) (see Figure S4).

Note that the interpretability of this comparison is limited for several reasons: Firstly, most phylogenetic methods such as PhyloWGS do not try to generalise trends in event orderings to datasets of many patients but rather infer orderings for individual samples only. Secondly, metMHN is designed to model the progression of simplified mutational profiles and does not claim to fully resolve evolutionary dynamics. The nuances that differentiate these related concepts are partly discussed in [3] and [1]. Furthermore, in general, phylogenetic methods can only temporally resolve relative timings between events that happened after clonal fixation of the most recent common ancestor. Thus, the signal they can provide is strongly driven by the most recent stages of progression before observation, while metMHN aims to equally give attention to orderings in early progression stages. Lastly, we have compared here inferences on two datasets which differ in qualities that might affect the comparison - such as the sequencing method and enrollment criteria.

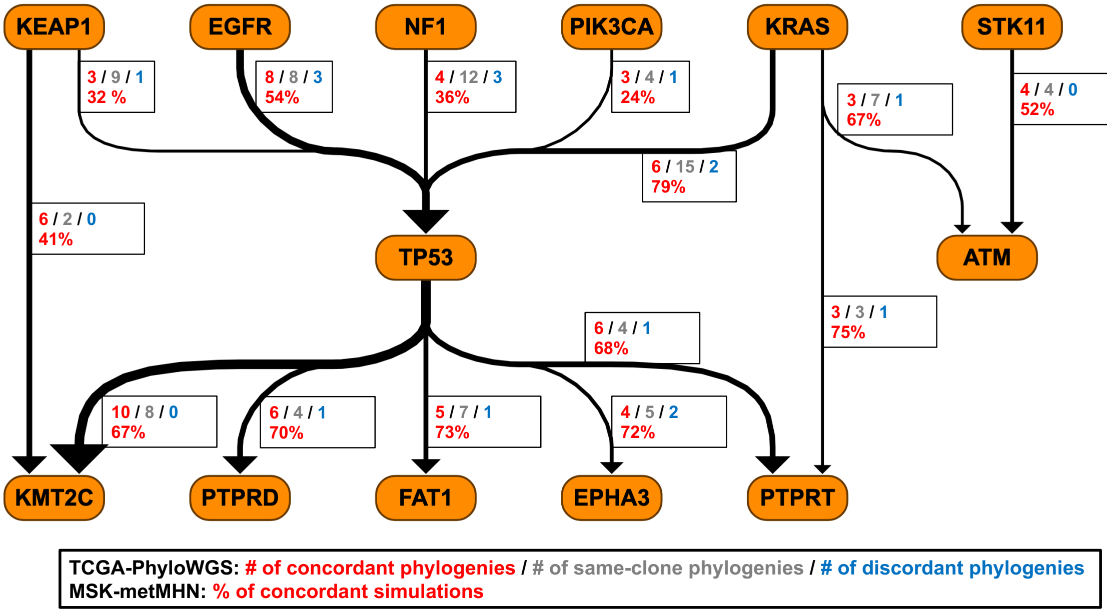

Figure S4: Trends in pairwise driver mutational event orderings as inferred by PhyloWGS and metMHN. For the 14 event pairs we analyse here, we have plotted the ordering that was more frequently observed in the PhyloWGS analyses of TCGA data. Line widths scale with the count of concordant phylogenies. In the respective text boxes, we further show the count of discordant and same-clone phylogenies as well as the ordering probability as derived from metMHN.

4 Supplementary plots

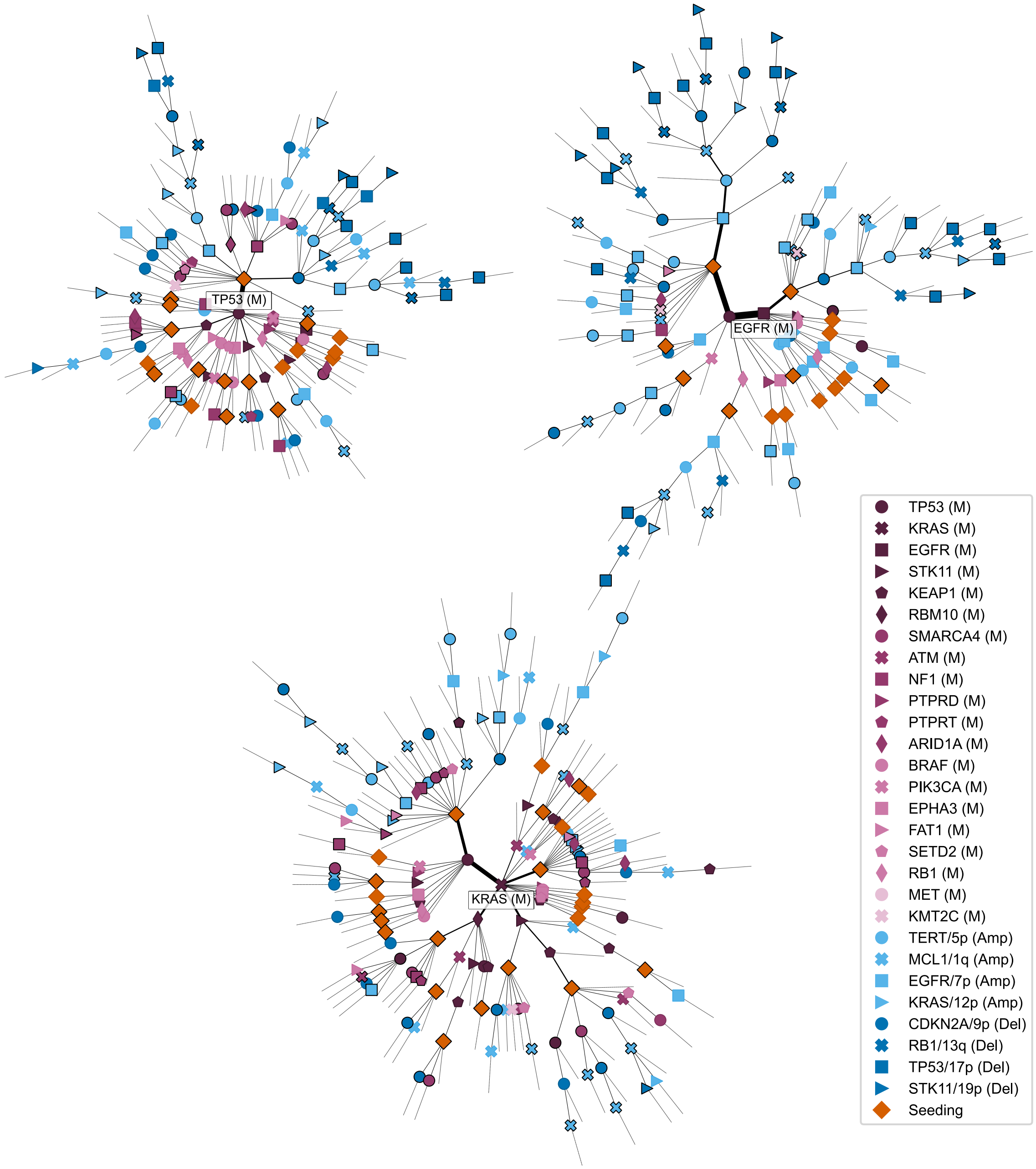

Figure S5: Most probable event orderings for observed metastases genotypes as inferred by metMHN, stratified by TP53 (M) (left), KRAS (M) (bottom) and EGFR (M) (right) as their first event. Each branch extending out from a tree's root represents a group of metastases for which the events were inferred to occur in the order of the branch. Edge widths scale proportionally to the dataset's count of metastases commencing with that particular sequence of events, and branches are trimmed at an edge threshold of 3. Black-bordered nodes indicate observed genotypes.

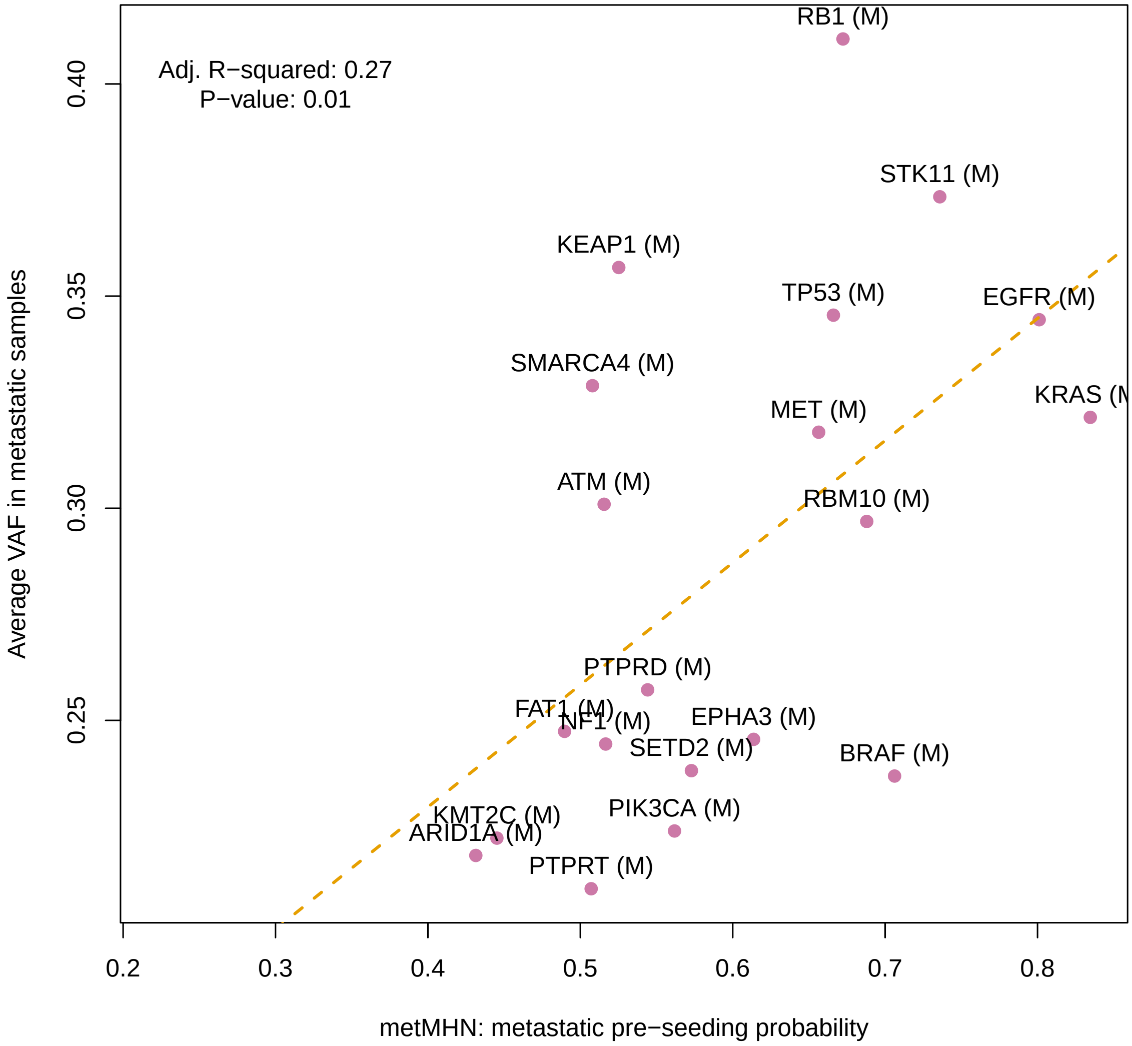

Figure S6: Metastasis pre-seeding probabilities for each mutational event, plotted against the average variant allele frequencies in copy number neutral cases for the respective event. A linear regression model was fit to evaluate the association, with an adjusted R-squared value of 0.27 and a p-value of 0.01.

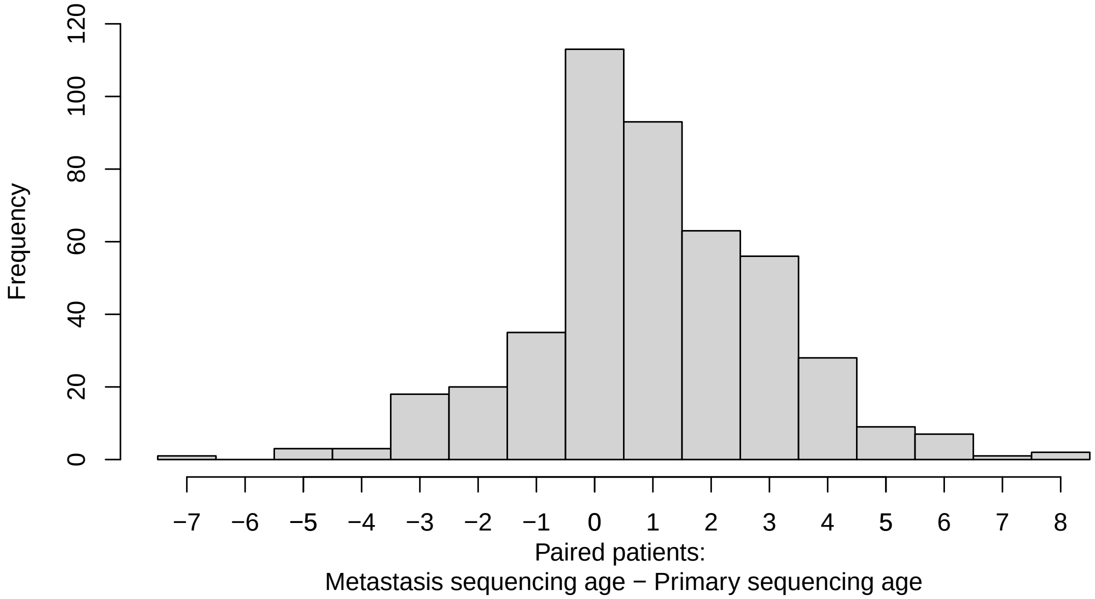

Figure S7: Distribution of sampling age differences for metastasis and primary tumor pairs in 452 patients for which both were available. Specifically, here we subtracted the integer patient age at which the sequencing of the primary tumor was reported from the integer patient age at which the sequencing of the metastasis was reported. For a single paired patient included in our analysis, both values were missing.

References

[1] Alves, J.M., Prieto, T. and Posada, D. Multiregional tumor trees are not phylogenies. *Trends in Cancer*, 3(8):546–550, August 2017.

[2] Deshwar, A.G. et al. Phylowgs: Reconstructing subclonal composition and evolution from whole-genome sequencing of tumors. *Genome Biology*, 16(1), February 2015.

[3] Diaz-Uriarte, R. A picture guide to cancer progression and monotonic accumulation models: evolutionary assumptions, plausible interpretations, and alternative uses, 2023.

[4] Gillespie, D.T. Exact stochastic simulation of coupled chemical reactions. *The Journal of Physical Chemistry*, 81(25):2340–2361, Dec 1977.

[5] Raynaud, F. et al. Pan-cancer inference of intra-tumor heterogeneity reveals associations with different forms of genomic instability. *PLOS Genetics*, 14(9):e1007669, September 2018.
